# Supplementary material for: Accuracy of real-time multi-model ensemble forecasts for seasonal influenza in the U.S
Source: PLoS Comput Biol. 2019 Nov 22;15(11):e1007486. doi: 10.1371/journal.pcbi.1007486 (PMC6897420; doi:10.1371/journal.pcbi.1007486)
Supplement: Attachment — Submitted filename: 201908-ploscompbio-response.docx (1).pdf [file pcbi.1007486.s002.pdf]

Response to reviewers for PCOMPBIOL-D-19-01044
"Accuracy of real-time multi-model ensemble forecasts for seasonal influenza in the U.S."
Reich et al.

Responses to reviewers are highlighted in **boldface blue text**.

We note that due to a request from the publications team at PLOS, we have removed panel B of Figure 1 due to a licensing issue with a previous publication from our group.

Reviewer #1: Reich and colleagues describe the development and validation of a multi-model ensemble approach for flu forecasting. This work has arisen out of a collaborative network of groups participating in the CDC's flu forecasting challenge. It is hard to find much to fault with this study. The paper is clearly written, and the analysis plan and execution are rigorous and well-thought out. This study provides a gold-standard for how forecasting work should be performed, with clear, pre-specified outcomes, extensive development with cross validation, and an out-of-sample, real-time test of the method. I have only minor comments to help with the interpretation of some of the results.

We thank the reviewer for the compliments on the importance and robustness of this manuscript.

1. I understand that the authors were constrained in the metrics that were reported based on the criteria for the CDC's contest. However, in some instances, it would be easier to interpret if the results were presented differently. For instance, the authors demonstrate that the TTW model performs better than the equal weight model. But it is difficult to evaluate from the forecast scores whether these improvements are meaningful. Expressing some of the results in terms of the units of measurement might be helpful. For instance, the estimate of peak week was off by an average of X weeks for TTW compared with Y weeks for equal-weighting.

We agree that the "forecast score" we present does not provide a single number that can be interpreted on the scale of weeks or incidence level. This is due to the fact that the forecasts are probabilistic by design and are evaluated as such by CDC. The forecast scores are the only metrics the CDC consults when determining overall forecast accuracy, so our pre-specified analyses did not include additional point-estimate analyses as suggested by the reviewer. We do provide an analysis of point estimate error in the supplement. In response to this comment and a related comment (#2 from Reviewer 2), we have clarified the distinction between point and probabilistic forecast evaluation and also added additional interpretation of the average bias in the results section.

The following was added to the Discussion section:
"Formally measuring the quality of forecasts is challenging and the choice of metric can impact how models are constructed. Following the FluSight Challenge guidelines, we used a probabilistic measure of forecast accuracy, the modified log score, as our primary tool for evaluating forecast accuracy. We also assessed point estimate accuracy as a secondary

measure (see Appendix). [...] In the case of the FluSight Network forecasts, the CDC has prioritized accuracy in a probabilistic sense over point-estimate accuracy."

The following was added to the Results section:
"For example, during the scoring period of interest across all regions in the 2017/2018 season, the FSNetwork-TTW model's point estimates for season onset were on average less than half a week above the true value (average bias = 0.38 week) and for 1-week ahead ILI the estimates were underestimated by less than one-quarter of a percentage point (average bias = -0.23 ILI%)."

2. Is there a way to visualize/summarize how the forecast accuracy for the different models changes throughout the season as data accrue for other forecasting targets (similar to what is done in figure 6 for peak week)?

Thanks for this suggestion. We added a figure to the supplement comparing the chosen ensemble to the simpler equal-weighted ensemble and the seasonal average model. We included the following sentence in the Results section of the main text:
"The FSNetwork-TTW model consistently outperformed a simpler ensemble model and the seasonal average model across all weeks of the 2017/2018 season (see Appendix)."
The new supplemental figure is shown below:

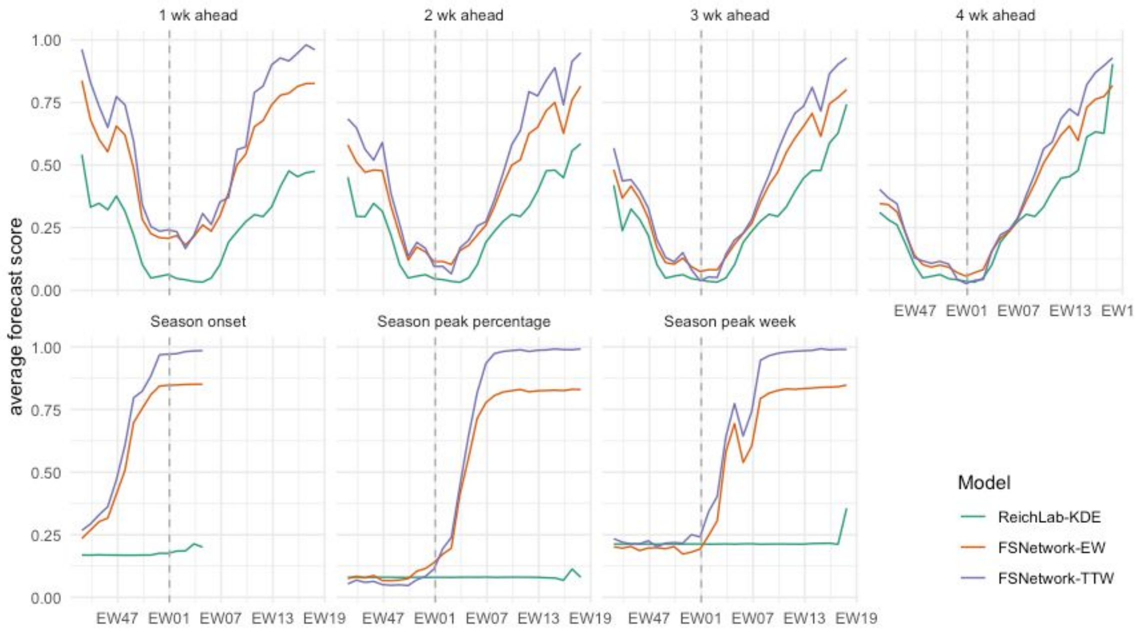

3. Given that the ensembles performed worse than the prior-season average for predicting peak intensity early in the season (Fig 6), would there be any benefit to including the prior years average itself in the ensemble?
Two "historical average" models (ReichLab-KDE and Delphi_EmpiricalTrajectories) are included in the component models available to the ensemble. These models are available to the ensemble for inclusion, however, the ensembles assign these models negligible weight.

We added a sentence to clarify this model was included as a component model in 'Methods > Ensemble components' section:
"Two components were constructed to represent a seasonal baseline based on historical data only."

We also added the underlined phrase in the 'Results > Choice of ensemble model based on cross-validation' section:
"The pre-specified ensemble approaches all relied on taking weighted averages of the component models, including two seasonal baseline components, using a predictive density stacking approach (see Methods)."

4. It seems that the ensemble weights here do not vary through the season and are based only on the cross-validation period. Would there be any benefit to using the cross-validation weights as a starting point and then allowing the ensemble weights to vary each week as the data accrue?
This is a good idea, and is the topic of ongoing research in our group. We have provided a citation to preliminary work (McAndrew and Reich, 2019) in the discussion section.

5. Could the authors comment on the criteria chosen by CDC for evaluating accuracy and precision as well as the forecasting targets and whether there are modifications that they would suggest based on their experience?
Yes, this is an excellent question, and actually the topic of a recent letter and response to the FluSight Network's first paper published in PNAS in January. (The letter and response have been accepted and are due to appear by the end of September 2019). We have added a brief summary of this issue in the discussion section, along with some new citations:

"It has been shown that the modified log score (i.e. multiple bins considered accurate) used by the CDC is not strictly proper and could incentivize forecasting teams to modify forecast outputs if their goal was only to achieve the highest score possible. (Gneiting 2007, Bracher 2019) Forecasts in the FluSight Network were not modified in such a way. (Reich 2019) Most component forecasts were optimized for the proper log-score (i.e. single bins considered accurate) while the FluSight Network ensembles were optimized to the modified log score. By using single bin scoring rules to evaluate forecasts, practitioners could ensure that all forecasts were optimized with the same goal in mind."

Reviewer #2: In this article, the authors demonstrate that a multi-model ensemble forecast was able to provide accurate forecasts of influenza-like illness activity over the course of the 2017/18 United States influenza season. They describe how a range of multi-model ensembles were created and trained on past years' data, how the best-performing ensemble was identified, and then entered into the CDC FluSight challenge. This ensemble not only out-performed each of the individual models in the ensemble, but also came second overall in the challenge, despite the 2017/18 season being highly unusual. These kinds of collaborative efforts and methodological developments are critical if such forecasts are to become a part of routine public health surveillance.

We agree, and appreciate the reviewer articulating the value of these collaborative translational research efforts.

Comments:

1. The motivation for selecting only one ensemble model as the official FluSight Network entry in the 2017/18 challenge is perfectly reasonable and pragmatic.

But it would be great to see even a cursory comparison of the selected ensemble model (FSNetwork-TTW) and the four other ensemble models (FSNetwork-EW, FSNetwork-CW, FSNetwork-TW, FSNetwork-TRW) for 2017/18, even though those other models weren't officially entered into the competition. In particular, it would be really interesting to see whether the performance similarities between these models in the training phase were also evident in such an unusual influenza season, or if the differences between these models conveyed any (dis)advantages in this scenario.
In addition to being reasonable and pragmatic, as the reviewer suggests, this choice was dictated by our pre-specified analysis plan and our desire to present an honest prospective comparison of forecast models based on the single model that we selected based on that analysis plan.

We added a figure and interpretation describing the comparison the reviewer requested in the supplement (section 4, Figure 5). Figure shown below:

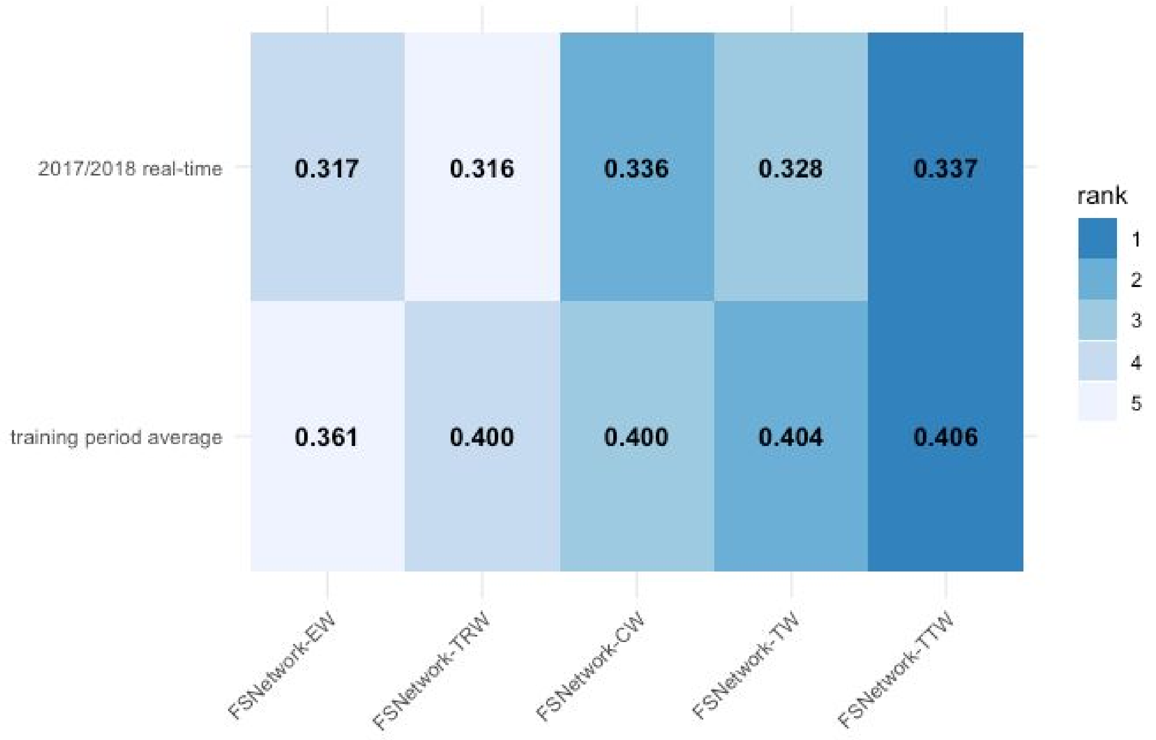

2. In the result section (page 8, lines 179-182) the authors state:

"Overall, the FSNetwork-TTW model ranked second among selected models in both RMSE and average bias, behind the LANL-DBM model (see Appendix), suggesting that using separate weighting schemes for point estimates and predictive distribution may be valuable."

This gave me a moment's pause. The reasoning is explained in more detail in section 2.1 of the supplementary material, and some of this detail could be included in the main text. Perhaps just a reminder that ensemble weights were chosen to maximize log scores, rather than point-estimate errors, would be sufficient.

As part of our response to Reviewer #1, comment 5, the following text has been added:
"[...] the FluSight Network ensembles were optimized to the modified log score."

3. In figure 4, cells with dark blue background could have values shown in white text or a light color, rather than black text, to make it easier to read. This also applies to Figure 1 in the supplementary material.

We thank the reviewer for pointing this out. Rather than have the color of the text change (which we thought might be confusing for readers) we opted to change the color scale so that the darkest blue color is now not as dark and the black text can be read more easily.

4. In the methods section (page 15, lines 388-389) a reference is missing:

"Second, multi-model ensembles combine component models through techniques such as model stacking (see Section)."

We have modified this to refer to the Methods section generally.
